# Comments on “The Sweet Scent on Baby’s Breath?”

**DOI:** 10.1289/ehp.10993

**Published:** 2008-04

**Authors:** Ladd W. Smith, Daniel T. Salvito

**Affiliations:** Research Institute for Fragrance Materials, Inc., Woodcliff Lake, New Jersey, E-mail: lsmith@rifm.org

The stated mission of *Environmental Health Perspectives* (*EHP*) is

to serve as a forum for the discussion of the interrelationships between the environment and human health by publishing in a balanced and objective manner the best peer-reviewed research and most current and credible news of the field. (EHP 2008)

We would like to focus on the part of your mission statement, regarding “a balanced and objective manner.” For the third time in about as many years, we find ourselves writing to *EHP* to correct inaccuracies in its reporting of the state of the science regarding fragrance ingredients and our understanding of their fate and effects in the environment and human health.

Your news item “The Sweet Scent on Baby’s Breath?” (Potera 2007) is yet another example of information conveyed by your journal in a manner that is neither balanced nor objective. For example, you quote the work of Luckenbach and Epel (2005), yet omit the clarifications from the Research Institute for Fragrance Materials (RIFM) that *EHP* published in a follow-up letter to the editor (Salvito 2005).

You continue to allow reiteration of the inaccurate statement that there is little known about the polycyclic musks, when in fact a robust body of scientific studies published in a number of peer-reviewed journals are in fact available, and have been used to support a thorough evaluation in Europe of the risks of these ingredients to human health and environment.

6-Acetyl-1,1,2,4,47-hexamethyltetraline (AHTN) and hexahydro-hexamethyl-cyclopenta (γ)-2-benzopyran (HHCB) have been assessed by the Scientific Committee on Cosmetic and Non-Food Products (SCCNFP 2002a, 2002b) of the European Union and were determined to be safe to human health for their use in cosmetic products. This was noted by Salvito (2005) in his response to the work of Luckenbach and Epel (2005). These ingredients have also been evaluated by the European Chemicals Bureau (ECB) for determination of their environmental hazards [persistence, bioaccumulation, and toxicity (PBT)], and the ECB has determined that these materials are not PBTs (European Chemical Bureau 2004). In addition, the SCCNFP issued favorable opinions on both AHTN and HHCB, finding them safe for use in cosmetic products (SCCNFP 2002a, 2002b). Further, there have been > 40 publications in peer-reviewed scientific journals pertaining to the human health and environmental safety of polycyclic musks (references available upon request).

As a peer-reviewed journal whose stated mission is to present the best science in an objective manner, we are disappointed with your continued lack of objectivity and inability to collect the necessary information to present a true perspective of the science. It would appear that your news staff needs to perform more thorough research in preparing their reports and that your peer-review process may be incomplete.

The RIFM, a nonprofit organization whose research is governed by an independent expert panel, was established to provide the research and testing necessary to assure the safety of ingredients used in the creation of fragrances. The RIFM has been in existence for > 40 years and has well-established relationships with academia; it is also well known among many regulatory agencies around the world for publishing its work in the peer-reviewed literature. Our organization, as well as others from our industry, are listed on the U.S. Environmental Protection Agency’s website under related links (U.S. EPA 2007).

## Editor’s note

Forum articles are short and cannot be all-inclusive of a topic. “The Sweet Scent on Baby’s Breath?”[Environ Health Perspect 115:A491 (2007)] focused on the presence of polycyclic musks in breast milk in the United States, which had never been measured before the study by Kannan and colleagues [Environ Sci Technol 41(11):3815–3820 (2007)]. That said, Smith and Salvito are correct that comment from an industry source should have been included in this article.

*Both researchers interviewed for this article [Environ Health Perspect 115:A491 (2007)] expressed concern about the impact of long-term bioaccumulation of polycyclic musks, as well as the lack of full understanding thereof. This concern is shared by others in the field; for example, the most recent Lake* Michigan Lakewide Management Plan *(http://www.epa.gov/lakemich/2006/index.html) includes six polycyclic musks—including AHTN and HHCB—on a watch list of pollutants to be reviewed in 2008. As researchers look at different end points (such as efflux transporters) or sentinel species (such as mussels), new data will continue to emerge that warrant further investigation, as well as reporting.*
